# The unity and the stability of human behavior. An interdisciplinary approach to habits between philosophy and neuroscience

**DOI:** 10.3389/fnhum.2014.00607

**Published:** 2014-08-11

**Authors:** José A. Lombo, José M. Giménez-Amaya

**Affiliations:** ^1^School of Philosophy, Pontifical University of the Holy CrossRome, Italy; ^2^Research group Science, Reason and Faith (CRYF), University of NavarrePamplona, Spain

**Keywords:** habit, learning, memory, basal ganglia, reason, plasticity

## Introduction

The study of learning and memory through routines is acquiring a growing interest in the present neuroscience. Many works focusing on “habit learning” highlight the relevance of such studies and much weight is given to it in the understanding of individual's behavior. The aim of this paper is to connect the concept of habit that arises from a neurobiological viewpoint and from a philosophical one. This will require a precise terminological distinction and connection between the two fields from an interdisciplinary approach.

One of the most recent studies of the use of the term “habit” in neuroscience is the review of Carol Seger y Brian Spiering, published in Frontiers in Systems Neuroscience in 2011. In this work, the authors make a historical evaluation of expressions “habit” and “habit learning” in these terms: « “Habit” roughly corresponded to the resulting motor behavior […], and habit learning to acquisition of these behaviors in an instrumental learning context. » “Habit” presents therefore these characteristics: inflexible, slow or incremental, unconscious, automatic and insensitive to reinforcer devaluation.

On the other hand, philosophical concept of habit (hexis, habitus) is not only—nor even mainly—related to a repetitive behavior, but to the control or “possession” of one's action (se habere ad). According to this, “habitus” is closer to the concept of “quality” or “skill” than to that of “stereotype” and appears as a vehicle of free will.

Apparently both approaches, neurobiological and philosophical, seem different and unconnected. Nevertheless, a possible bridge between them is the consideration of habit as “stable disposition for self-development.” In order to illustrate this statement, we will follow several steps throughout the paper. First, we will expose how the concept of life implies a kind of self-activity and self-control, which requires either stability and change at different levels. Second, we will deal with the neurobiological understanding of stable behavior as seen in neurobiological processes such as learning and memory, “habit learning,” etc. And thirdly, we will explain how the philosophical concept of “habit” corresponds to dispositive quality as control of one's action. Finally, we will try to integrate the two perspectives.

## Habits as stable control of actions

From a philosophical point of view, life consists in the activity of an individual over itself (self-activity, self-control). A living being is capable of actions whose outcome doesn't remain only outside, but inside the living being itself. This kind of activity implies a general scheme of “feedback” and corresponds with the Aristotelian concept of “praxis,” as different to the concept of “poiesis.” “Praxis” is an activity whose aim is the activity itself, and so its outcome remains in the individual (Metaphysics, IX, 6, 1048 b 18–35; Aristotle, 1924). Instead, “poiesis” is an activity that produces something different from the action itself and so it has an external outcome (Nicomachean Ethics, VI, 4, 1140a 1–6; Aristotle, 2011). Even though poiesis and praxis are different, they are not necessarily separable, but continuously interwoven in living beings endowed with knowledge. Life as a whole is praxis, but particular life activities include both poiesis and praxis (Aristotle, Politics, I, 2, 1254 a 7–8, Aristotle, 1990; Vicente Arregui and Choza, 1991).

In the interaction of poiesis and praxis, the living being not only maintains its own structure, but it progressively develops it. In general, this development consists in the extension or amplification of one's own physical structure. Nevertheless, in the case of living beings endowed with knowledge, development has also an intensive dimension, as they can acquire new capabilities through their interaction with other beings. This intensive development can be understood as “learning” or “accumulated experience.” It results from single actions, but it differs from them as an acquired and stable capability. Aristotle called that capability “habit” (hexis, habitus) and understood it as making the subject of it able to perform new actions (Nicomachean Ethics, II, 4, 1106 b 36; Aristotle, 2011).

## Neurobiological understanding of stable behavior: learning and memory, “habit learning,” etc.

On the part of empirical research, modern psychology has studied the concept of “habit” quite in detail. The context of it has been the study of learning and, more in general, of animal behavior: see, for example, James (1890) and Watson (1919). This perspective has gained in depth thanks to the development of cognitive experimental psychology and the studies on learning and memory during XX century (Bernácer and Giménez-Amaya, 2013).

Since the second half of the 50's, neuroscientific studies showed the progressive implication of structures of the temporal lobe in memory and in learning (Scoville and Milner, 1957; Bernácer and Giménez-Amaya, 2013). Those studies defined a distinction between an explicit memory and an implicit one: in the former, cortical structures are mostly involved, mainly medial portions of temporal lobe; in the latter, some subcortical structures stand out, which belong to the basal ganglia.

In sum, there has been a progressive separation of two neurobiological processes related to memory. On the one hand, some mnesic processes reveal learning as related to processes of plasticity, which imply a high cortical activity (explicit memory). On the other hand, other processes evince learning as the stabilization of patterns of behavior—mainly motor—, in which some subcortical structures intervene, as the aforementioned basal ganglia (implicit memory).

The concept of “habit learning” was introduced in cognitive neuroscience through these premises. According to Seger and Spiering (2011): “The concept of habit learning has developed through the fruitful interaction of researchers in several intellectual domains, including animal learning, cognitive psychology, cognitive neuropsychology, and behavioral neuroscience.” In large measure, the concept of “habit learning” has been related to subcortical structures of basal ganglia and, therefore, to processes of learning involved in implicit memory: see, for example, reviews of Seger and Spiering (2011) and Graybiel (2008).

Basal ganglia are structures strongly connected among themselves, with a fundamental role in the organization of complex circuits of cortical and subcortical feedback (Mengual et al., 1999; Obeso et al., 2002; Packard and Knowlton, 2002; Lanciego et al., 2012). Two traits make them especially relevant to study processes of learning and memory. First, their neural circuits of feedback are much wider and more complex than what was originally thought. In fact, basal ganglia are not only related to motor system in itself, but they are also important as nodal points in broad neural networks, which integrate motor behavior with emotional and motivational life, particularly frontostriatal circuits and limbic areas: see, for instance, reviews of Haber and Rauch (2010) and of Hwang (2013). Second, they are privileged structures of central nervous system for the understanding, at a molecular and synaptic level, of the strong interaction between neurotransmitters and neuromodulators involved in networks of implicit memory (Kreitzer and Malenka, 2008). This permits to establish complex patterns of cellular integration and of relations of nervous cells among them.

These remarks show the significance of basal ganglia in the study of “habit learning.” This kind of learning has been described as “inflexible, slow or incremental, unconscious, automatic, and insensitive to reinforcer devaluation” (Seger and Spiering, 2011). Nevertheless, there is increasing evidence that, through their cortical and subcortical circuits, some degree of flexibility and control can be established (Smith and Graybiel, 2014). In fact, “habit learning” seems to be open to include instances of plasticity, learning and memory (Graybiel, 2008; Howe et al., 2011). As a result, several approaches to “habit learning” are increasingly seeing it as a balance between behavioral flexibility and fixity (Smith and Graybiel, 2014).

On one hand, some authors have regarded the idea of “habit learning” as the performance of an action, previously learned after many repetitions, in an unconscious manner, and whose execution is inflexible and independent to the outcome (Seger and Spiering, 2011; Bernácer and Giménez-Amaya, 2013). On the other hand, this perspective should be integrated with other view that recognizes sensitivity to the outcome and hence different levels of flexibility and feedback, allowing integrating changes onto behavioral processes or strategies. In this way, the whole system allows several levels of increase and development (Lombo and Giménez Amaya, 2013; Smith and Graybiel, 2014).

## Adaptation and change: stability vs. rigidity

From the mentioned approach, some opposition can be established between two ways of understanding “habit learning.” On the one hand, it appears as a rigid and stereotyped behavior (Seger and Spiering, 2011). On the other hand, it can be understood in a more open and flexible way, what allows the incorporation of phenomena of variability within a general scheme of control (Graybiel, 2008; Smith and Graybiel, 2014).

Deep in this opposition, we discover that the second view does not exclude the first one, but rather it presupposes it. In fact, a habit is not a mere automatism or a repetitive behavior, but a stable disposition for action (practical skill). The difference between habits and automatisms or simple routines is that the former give control over actions, while the latter don't (Nicomachean Ethics, II, 1, 1103 a 14-b 25; Aristotle, 2011). As a consequence, the stability of habits differs from the rigidity of automatisms.

Consequently, rigidity and the stereotyped character of “habit learning” should be understood as “stability” of behavior, rather than as an irremovable configuration of it. This is therefore a richer concept, from a semantic standpoint, and points out to a stable basic structure on which living being's behavior is organized in a flexible manner. This flexibility allows adaptation to new stimuli and development of new abilities. On the other hand, excessive inflexibility makes adaptation impossible and may lead to behavioral disorders, like obsessive-compulsive personality disorder, for instance (De Reus and Emmelkamp, 2012).

This neurobiological view of “habit learning” and recent experimental contributions—especially those of professor Graybiel—are consistent with the philosophical concept of “habit” in human being. This one is essentially based on two aspects: (a) the stable character of an acquired quality; and (b) the capacity for new actions that arises from that quality (Millán-Puelles, 2002).

In first place, habit is related to “having,” as the term indicates in its Latin original form (“habitus” comes from “habere,” to have). According to Aristotle, a subject may have other realities or may have itself as related to other realities (Nicomachean Ethics, II, 4, 1105 b 25–26; Aristotle, 2011). This “having himself” as related to something means actually “to be disposed in relation to something”: Aristotle's Metaphysics, V, 20; 1022 b 10–12 (Aristotle, 1924). For this author, habit (“hexis”) is not a simple reaction to the influence or activity of other subjects (he calls this influences “pathe,” passions). It is rather to “dispose himself” from that influence, acquiring a stable capacity to accomplish something in a way that becomes usual. A habit can be described therefore as a usual way of behaving, so that Aristotle refers to it also as a “second nature”: Aristotle's Categories, VIII 9 a 4 (Aristotle, 1930). Inasmuch as “habit” is not a simple reaction, but a stable disposition to action, it has been compared with cybernetic processes (Polo, 2002). This disposition, in fact, is stable and progressive, but not properly rigid.

## Conclusive remarks

As we have seen, neurobiological concept of “habit” is reflected in the so called “habit learning.” This implies two main aspects, that's to say, stability of behavior (that can be interpreted as “rigidity” or “stereotype”) and its flexibilization in front of new stimuli (Seger and Spiering, 2011; Smith and Graybiel, 2014). This is clearly verified in superior mammals, but in the case of human being we find a special richness in his behavioral response. Neurobiological ground of that higher development can be found in the remarkable growth of his cortical and subcortical networks (basal ganglia, among other structures), and in his extraordinary cellular and high synaptic variety (see for example, Nijhuis et al., 2013).

We can discover, in sum, a connection between neurobiological and philosophical standpoints. On one hand, “habit learning” implies a stabilization of neurobiological information that subsequently allows its storage and re-utilization in front of new stimuli. On the other, philosophical description of “habit” presents it as feedback of human activity. This feedback allows not only to keep our activities, but also to use them again in front of new phenomena, making possible continuity and articulation of experience.

### Conflict of interest statement

The authors declare that the research was conducted in the absence of any commercial or financial relationships that could be construed as a potential conflict of interest.
